# Deletion of the Prorenin Receptor from the Ureteric Bud Causes Renal Hypodysplasia

**DOI:** 10.1371/journal.pone.0063835

**Published:** 2013-05-21

**Authors:** Renfang Song, Graeme Preston, Atsuhiro Ichihara, Ihor V. Yosypiv

**Affiliations:** 1 Department of Pediatrics, Hypertension and Renal Center of Excellence, Tulane University School of Medicine, New Orleans, Louisiana, United States of America; 2 Department of Medicine, Institute of Endocrinology and Hypertention, Tokyo, Japan; UCL Institute of Child Health, United Kingdom

## Abstract

The role of the prorenin receptor (PRR) in the regulation of ureteric bud (UB) branching morphogenesis is unknown. Here, we investigated whether PRR acts specifically in the UB to regulate UB branching, kidney development and function. We demonstrate that embryonic (E) day E13.5 mouse metanephroi, isolated intact E11.5 UBs and cultured UB cells express PRR mRNA. To study its role in UB development, we conditionally ablated *PRR* in the developing UB (*PRR* ^UB−/−^) using *Hoxb7* ^Cre^ mice. On E12.5, *PRR* ^UB−/−^ mice had decreased UB branching and increased UB cell apoptosis. These defects were associated with decreased expression of *Ret*, *Wnt11*, *Etv4*/*Etv5*, and reduced phosphorylation of Erk1/2 in the UB. On E18.5, mutants had marked kidney hypoplasia, widespread apoptosis of medullary collecting duct cells and decreased expression of *Foxi1*, *AE1* and *H^+^-ATPase α4* mRNA. Ultimately, they developed occasional small cysts in medullary collecting ducts and had decreased nephron number. To test the functional consequences of these alterations, we determined the ability of *PRR* ^UB−/−^ mice to acidify and concentrate the urine on postnatal (P) day P30. *PRR* ^UB−/−^ mice were polyuric, had lower urine osmolality and a higher urine pH following 48 hours of acidic loading with NH_4_Cl. Taken together, these data show that PRR present in the UB epithelia performs essential functions during UB branching morphogenesis and collecting duct development *via* control of *Ret*/*Wnt11* pathway gene expression, UB cell survival, activation of Erk1/2, terminal differentiation and function of collecting duct cells needed for maintaining adequate water and acid-base homeostasis. We propose that mutations in *PRR* could possibly cause renal hypodysplasia and renal tubular acidosis in humans.

## Introduction

Congenital anomalies of the kidney and urinary tract (CAKUT) occur in 3–6 per 1000 live births and account for 31% of all cases of end-stage kidney disease (ESKD) in children in the United States [1]. All forms of CAKUT stem from abnormal kidney development [1], [2]. Branching morphogenesis of the ureteric bud (UB) is a key developmental process that directs organogenesis of the metanephric kidney [3], [4]. Terminal tips of branching UBs induce surrounding mesenchyme-derived nephron progenitors to differentiate into nephrons, thus forming the metanephric kidney [3], [4]. Following completion of UB branching, UB-derived collecting ducts undergo terminal differentiation- acquisition of distinct epithelial cell types that perform specialized functions [4], [5]. Notably, derangements in UB morphogenesis or UB cell differentiation result in CAKUT and distal renal tubular disorders, respectively [3]–[8].

The PRR is the cell-surface receptor for renin and prorenin, and an accessory subunit of the vacuolar proton pump H^+^-ATPase [9]–[11]. In the adult rat collecting duct, PRR is most abundant at the apical surface of type α intercalated cells (α-ICs) where it colocalizes with the H^+^-ATPase and may be activated in a paracrine fashion by prorenin or renin released by adjacent principal cells [12], [13]. Moreover, H^+^-ATPase is required for the activation the extracellular signal-regulated kinase 1/2 (Erk1/2) induced by prorenin or renin in the collecting duct cells [12]. Critical role for H^+^-ATPase in development is evident from the observation that mutations in the genes encoding specific subunits of H^+^-ATPase in mice result in embryonic lethality or metabolic acidosis [14], [16]. Given that pharmacologic inhibition of Erk1/2 decreases UB branching [17], disruption of PRR signaling in the UB may lead to aberrant UB morphogenesis and renal collecting system development. In addition, PRR may promote differentiation of H^+^-secreting intercalated cells in the developing collecting duct.

Here, we tested the hypothesis that targeted inactivation of the *PRR* in the UB epithelia in mice is essential for UB branching morphogenesis and collecting duct development. We demonstrate that *Cre*-mediated inactivation of the *PRR* targeted to the UB disrupts UB branching, reduces the number of nephrons and causes renal hypodysplasia. Reduced phosphorylation of Erk1/2 in the UB, widespread apoptosis of UB and medullary collecting duct cells, aberrant expression of *Ret*/*Wnt11* UB morphogenetic program genes and collecting duct cell differentiation markers such as Foxi1, AE1, H^+^-ATPase and Aqp2 is observed in mutant kidneys. These findings demonstrate that PRR present in the UB epithelia performs essential functions during UB branching morphogenesis and collecting duct development *via* control of *Ret*/*Wnt11* pathway gene expression, UB cell survival, activation of Erk1/2 signaling and differentiation of collecting duct cells involved in acid-base homeostasis and concentration of the urine.

## Materials and Methods

### Generation of UB-specific *PRR*-knockout Mice

*PRR*-floxed mice were provided by Dr. Atsuhiro Ichihara (Keio University, Tokyo, Japan) [10]. To delete *PRR* conditionally in the ureteric bud (UB), we used the *Hoxb7* ^Cre^ transgene, which drives Cre expression in the Wolffian duct and UB epithelium from E9.5 onwards [18]. The resulting *Hoxb7* ^Cre+^/*PRR* ^flox/flox^ mice represent UB-specific *PRR*-knockout mice (*PRR* ^UB−/−^). Control mice consisted of *Hoxb7* ^Cre−/^ *PRR* ^flox/flox^ and *Hoxb7* ^Cre−/^ *PRR* ^flox/+^ (*PRR* ^UB+/+^) littermates. UB-specific knockout of *PRR* was confirmed by qRT-PCR analysis which revealed an 80% decrease in PRR mRNA levels in E11.5 intact isolated UBs (iUBs) from *PRR* ^UB−/−^ compared with *PRR* ^UB+/+^ mice (0.19±0.03 vs. 1.0±0, p<0.001). On P1, UB-specific knockout of PRR was confirmed by double immunostaining of PRR and Aqp2, which revealed no PRR expression in the collecting duct in *PRR* ^UB−/−^ mice. All experiments involving mice were approved by Tulane Institutional Animal Care and Use Committee. All animal work involved in the generation of *in situ* hybridization probes by Dr. Jing Yu was approved by the University of Virginia Animal Care and Use Committee.

### Reverse-transcription Polymerase Chain Reaction (RT-PCR) and Quantitative RT-PCR

RT-PCR was utilized to determine whether cultured UB cells and E11.5 iUBs express PRR mRNA using PRR-specific primers: sense- 5′-CACATTGCGTCAG-CTCCGTAA-3′; antisense- 5′- CTCACCAGGGATGTGTCGAAT-3′. UB cells (a kind gift from Dr. J. Barasch, Columbia University, New York, NY) were initially obtained from microdissected ureteric buds of an embryonic day 11.5 mouse transgenic for simian virus 40 (SV40) large T antigen (Immorto-mouse, Charles River) [19]. qRT-PCR was performed to confirm elimination of *PRR* from E11.5 iUBs of *PRR* ^UB−/−^ mice. iUBs from *PRR* ^UB−/−^ and control mice were isolated as previously described Song [20]. qRT-PCR was performed in the Mx3000P equipment (Stratagene, La Jolla, CA) using MxPro QPCR software (Stratagene) as previously described [21]. mRNA was extracted from snap-frozen E11.5 iUBs, E12.5 and E18.5 *PRR* ^UB−/−^ and control kidneys (E11.5 iUBs and E12.5 kidneys were pooled, E18.5- n = 3 mice per group). The quantity of each target mRNA was normalized by that of GAPDH mRNA expression. RNA samples were analyzed in triplicates in each run. PCR reaction was performed twice.

### Immunohistochemistry and Histopathology

Kidneys were fixed in 4% PFA at 4°C and paraffin embedded. Immunostaining was performed by the immunoperoxidase technique using 4-µm sections with Vectastain Elite kit (Vector Laboratories, Burlingame, CA). Primary antibodies included anti-PRR (1∶200, Santa Cruz), anti-Aqp2 (1∶200, Santa Cruz), anti-H^+^-ATPase α4 (1∶1000) [22], rabbit polyclonal anti-AE1 (1∶200), anti-phospho-Erk1/2 (1∶200, Cell Signaling) and anti-Lotus Tetragonolobus Lectin (LTL) (1∶400, Vector Laboratories). Whole intact E12.5 metanephroi from *PRR* ^UB−/−^ and control mice (n = 6 mice and n = 12 kidneys per genotype) were processed for the whole mount immunofluorescence using anti-cytokeratin (1∶200, Sigma) and anti-WT1 (1∶100, Abcam) antibodies and the number of UB tips was counted. For immunofluorescence studies, secondary antibodies were detected with Alexa Fluor dyes (Invitrogen). Specificity of immunostaining was documented by the omission of the primary antibody. Left kidneys from P1 *PRR* ^UB−/−^ and *PRR* ^UB+/+^ mice (n = 3 mice per group) were cut in the longitudinal midplane, processed through the paraffin, and embedded on the cut surface. Kidneys were sectioned at 4-µm and stained with hematoxylin and eosin. The number of nephrons in each of 3 consecutive sections adjacent to the longitudinal midplane was counted and the mean number of nephrons per section per kidney was calculated. To determine the number of WT1-positive structures, we examined the intensity of WT1 immunostaining (1∶100, Abcam) in P1 kidney sections (n = 3 mice per group) using Slide book 4.0 software (Intelligent Imaging Innovations, Denver, CO). Total number of H^+^-ATPase α4-expressing cells in E18.5 collecting ducts was counted (n = 3 mice per genotype, 3 sections per kidney, 10 collecting ducts/section). More medullary domains of the kidney were chosen to count the number of H^+^-ATPase-expressing cells. All counts were performed in a blinded fashion.

### In situ Hybridization (ISH)

Section ISH was performed on E14.5 and E18.5 *PRR* ^UB−/−^ and control kidneys as previously described [21]. Mouse full length probes for *Foxi1* and *Aqp2* were a kind gift from Dr. Jing Yu (University of Virginia) [23]. 4 embryonic kidneys per group per probe were examined.

### Cell Proliferation and Apoptosis Assays

Cell proliferation and apoptosis was examined in E13.5 and E18.5 kidney sections from *PRR* ^UB−/−^ and control mice (n = 3 mice per genotype, 3 sections per kidney) as previously described [21]. Cell proliferation and apoptosis was assessed throughout the entire UB epithelium on E13.5. Anti-phospho-histone H3 (pH3) and anti-cleaved caspase-3 antibodies were used (Cell Signaling, Danvers, MA; 1∶50). UBs and collecting ducts were visualized with anti-cytokeratin antibody (1∶200, Sigma). The number of proliferating and apoptotic cells in the UB/collecting duct epithelia was normalized to the total number of DAPI-positive (Invitrogen) cells in each kidney section. The number of DAPI-positive cells was determined by Image J software (NIH).

### Measurement of Serum Creatinine, Urine Volume, Osmolality and pH

*PRR* ^UB−/−^ and control mice (n = 4 mice per genotype) were housed in metabolic cages (Hatteras Instruments, Cary, NC), fed a standard chow and allowed free access to tap water. 24-hour urine was collected at baseline and after administration of NH_4_Cl (0.8 g/kg body weight in drinking water) for 48 hours and was processed immediately for pH analysis by the pH meter (Fisher Scientific) [8]. Plasma creatinine was measured by HPLC with picric acid (Jaffe method).

### Western Blot Analysis

E12.5 kidneys from *PRR* ^UB−/−^ and control mice (n = 4 kidneys per genotype per one pooled sample, n = 3 pooled samples per genotype) were pooled and homogenized in cold lysis buffer containing a cocktail of enzyme inhibitors. Proteins (60 µg/lane) were processed for Western blot analysis as previously described [24]. After blocking nonspecific binding, the membranes were incubated with the phosphospecific anti-phospho-Erk1/2 antibody (1∶200, Cell Signaling). After stripping, membranes were reprobed with anti-total Erk1/2 antibody (Cell Signaling) to document equal protein loading. Immunoreactive bands were visualized using the enhanced chemiluminescence detection system (ECL, Amersham) as previously described [24].

### Statistics

Statistical analyses were carried out upon all biologic replicates with Student’s t test or a one-way ANOVA, followed by Bonferroni test. Data are presented as Mean±SEM. A p value of <0.05 was considered statistically significant.

## Results

### PRR Expression in Control and *PRR* ^UB−/−^ Developing Kidneys

**Figure 1:**
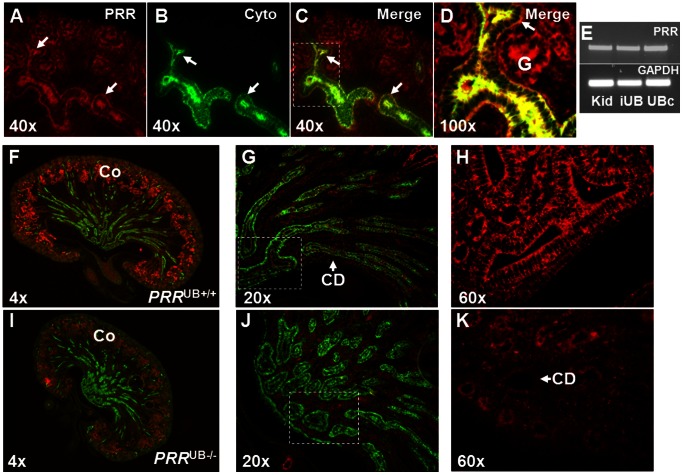
Immunolocalization of PRR protein in the mouse kidney on embryonic days E13.5 and P1. A–D: Sections were co-stained with anti-PRR (red staining) and anti-pancytokeratin (green staining) antibodies. PRR is detectable using antibody concentrations of 1/100 in the ureteric bud (UB) (arrows) and glomeruli (G). E: E13.5 wild-type mouse kidney (Kid), intact UBs (iUB) isolated from E11.5 wild-type mouse kidneys and immortalized UB cells (UBc) express PRR mRNA (521 bp). F–H: In *PRR* ^UB+/+^ kidney, PRR (red staining) is detected in the cortex (Co) and collecting ducts (CD). CDs are visualized with anti-Aqp-2 antibody (green staining). I–K: In *PRR* ^UB−/−^ kidney, PRR immunoreactivity is present in the cortex (Co) only and is not detected above the background level in Aqp-2-positive collecting ducts.

To determine the PRR expression during kidney development, we performed immunohistochemistry studies on sections of E13.5 and P1 mouse kidneys. Sections of E13.5 wild-type kidney reveal PRR immunostaining in the UB, nephron epithelia and in nascent glomeruli (Figure 1A–D). While *PRR* ^UB+/+^ mice expressed PRR in the collecting ducts and the mesenchyme on P1 (Figure 1F–H), kidneys of *PRR* ^UB−/−^ mice showed PRR labeling in the mesenchyme only and no expression in the collecting ducts (Figure 1I–K). RT-PCR demonstrated apparently similar expression levels of PRR mRNA in the whole intact E13.5 wild-type mouse kidney, in intact UBs isolated from E11.5 wild-type mouse kidneys, and in immortalized UB cells grown *in vitro* (Figure 1E). Thus, at early and later stages of metanephric development, PRR is expressed in both metanephric and UB lineages.

### *PRR* Deletion Results in UB Branching Defects, Downregulation of *GDNF*, *Ret* and its Target Genes

**Figure 2:**
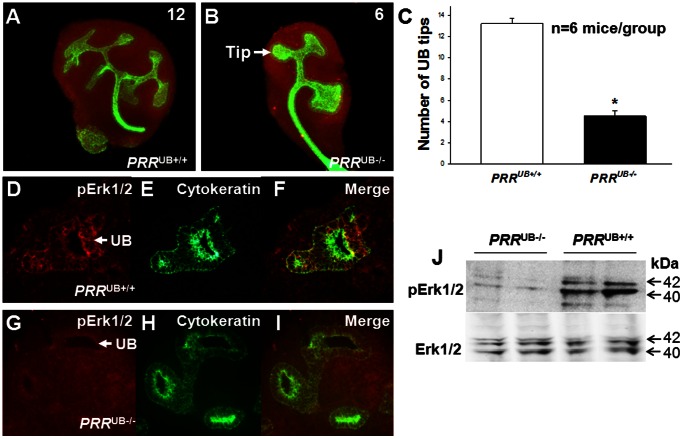
Effect of targeted genetic inactivation of the *PRR* in the ureteric bud (UB) on UB branching and Erk1/2 phosphorylation in E12.5 metanephroi. A, B: Metanephroi were co-stained with anti-pancytokeratin antibody to visualize the UB (green) and anti-WT1 antibody to visualize metanephric mesenchyme (red). The number of UB tips is reduced in mutant *PRR* ^UB−/−^ compared with *PRR* ^UB+/+^ kidneys. C: Bar graph showing the effect of *PRR* deletion in the UB on the number of UB tips. D–I: Sections of E12.5 kidneys show that phospho Erk1/2 (pErk1/2) immunostaining (red) is reduced in the UB of mutant (G, I) compared with control (D, F) mice. J. Whole E12.5 kidney lysates were subjected to Western blotting with anti-pErk1/2 antibody. After stripping, the membrane was reprobed with anti-total Erk1/2 antibody (Erk1/2). Erk1/2 phosphorylation appears to be reduced in *PRR* ^UB−/−^ compared with *PRR* ^UB+/+^ kidneys.

**Figure 3:**
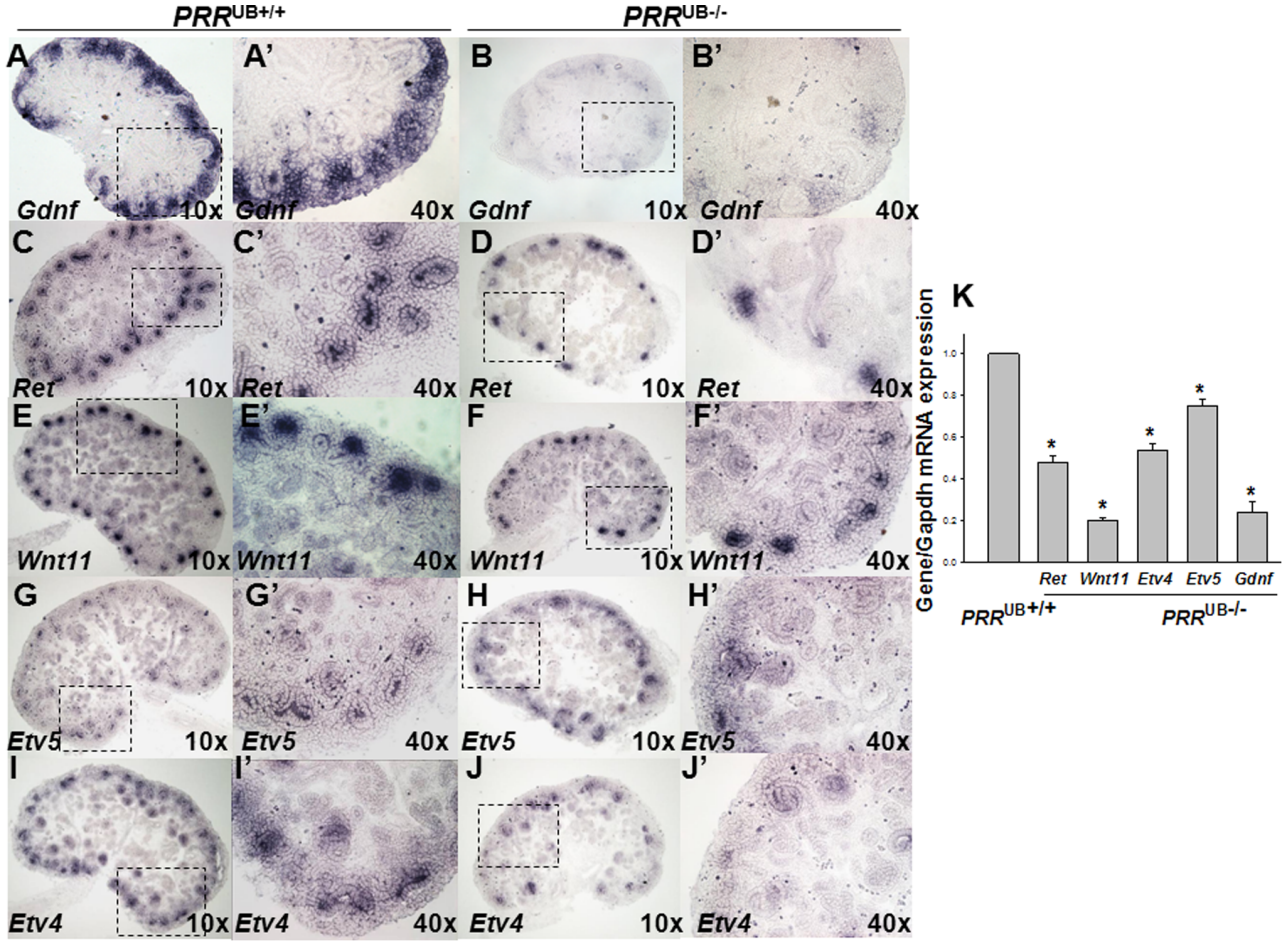
Effect of targeted genetic inactivation of the *PRR* in the UB on *Gdnf*, *Ret*, *Wnt11*, *Etv4* and *Etv5* mRNA expression in the mouse metanephros on E14.5. A–J’: Representative images of E14.5 section *in situ* hybridization. *PRR* ^UB−/−^ kidneys (B, D, F, H, J) are smaller and have decreased mRNA expression intensity compared with *PRR* ^UB+/+^ kidneys (A, C, E, G, I). High power images of areas shown by dashed line insets appear to show a reduction in *Gdnf* expression in the mesenchyme (B’ vs. A’) and of *Ret*, *Wnt11*, *Etv4* and *Etv5* expression at the tips of ureteric buds in *PRR* ^UB−/−^ (D’, F’, H’, J’) compared with *PRR* ^UB+/+^ kidneys (C’, E’, G’, I’), respectively. K: Bar graph showing decreased *Gdnf*, *Ret*, *Wnt11*, *Etv4* and *Etv5* mRNA levels in whole E12.5 *PRR* ^UB−/−^ metanephroi as determined by qRT-PCR. *p<0.001. *PRR* ^UB+/+^ value is defined as 1.

To assess the importance of PRR expression for UB and UB-derived collecting duct development, we examined whether targeted deletion of *PRR* from the UB disrupts UB branching. Anti-pancytokeratin antibody staining of whole intact E12.5 metanephroi showed a drastic reduction in the number of UB tips in mutants (4±0.6 vs. 13±0.5, p<0.001) (Figure 2A–C). Given that PRR activation normally triggers Erk1/2 phosphorylation in many cell types [25] and that pharmacologic inhibition of Erk1/2 inhibits UB branching [17], we investigated Erk1/2 phosphorylation in the UB of mutant and control kidneys. Immunostaining for phospho-Erk1/2 (pErk1/2) was reduced in the UB of *PRR* ^UB−/−^ compared with control E12.5 metanephroi (Figure 2D–I). These observations were confirmed by Western blot analysis, demonstrating decreased pErk1/2/total Erk1/2 ratios in *PRR* ^UB−/−^ compared with *PRR* ^UB+/+^ whole E12.5 kidneys (densitometric unit ratio: 0.37±0.04 vs. 0.69±0.03, p<0.001) (Figure 2J). To determine whether aberrant expression of *Gdnf*, its receptor *Ret* and *Ret* targets, *Wnt11*, *Etv4* and *Etv5*, could account for UB branching defects observed in *PRR* ^UB−/−^ kidneys, we examined *Gdnf*, *Ret*, *Wnt11*, *Etv4* and *Etv5* mRNA expression on E13.5 by whole-kidney qRT-PCR and on E14.5 by ISH. ISH demonstrated apparent reduction in *Gdnf*, *Ret*, *Wnt11*, *Etv4* and *Etv5* expression in *PRR* ^UB−/−^ compared to control kidneys (Figure 3). qRT-PCR demonstrated that *Gdnf*, *Ret*, *Wnt11*, *Etv4* and *Etv5* mRNA levels were lower in *PRR* ^UB−/−^ compared to control kidneys (Figure 3K). Thus, UB branching defects in *PRR* ^UB−/−^ mice appear do be due, in part, to reduced *Gdnf*/*Ret* expression and signaling *via Etv4/5* and Erk1/2.

### *PRR* Deletion Results in an Increased UB and Collecting Duct Cell Apoptosis

**Figure 4:**
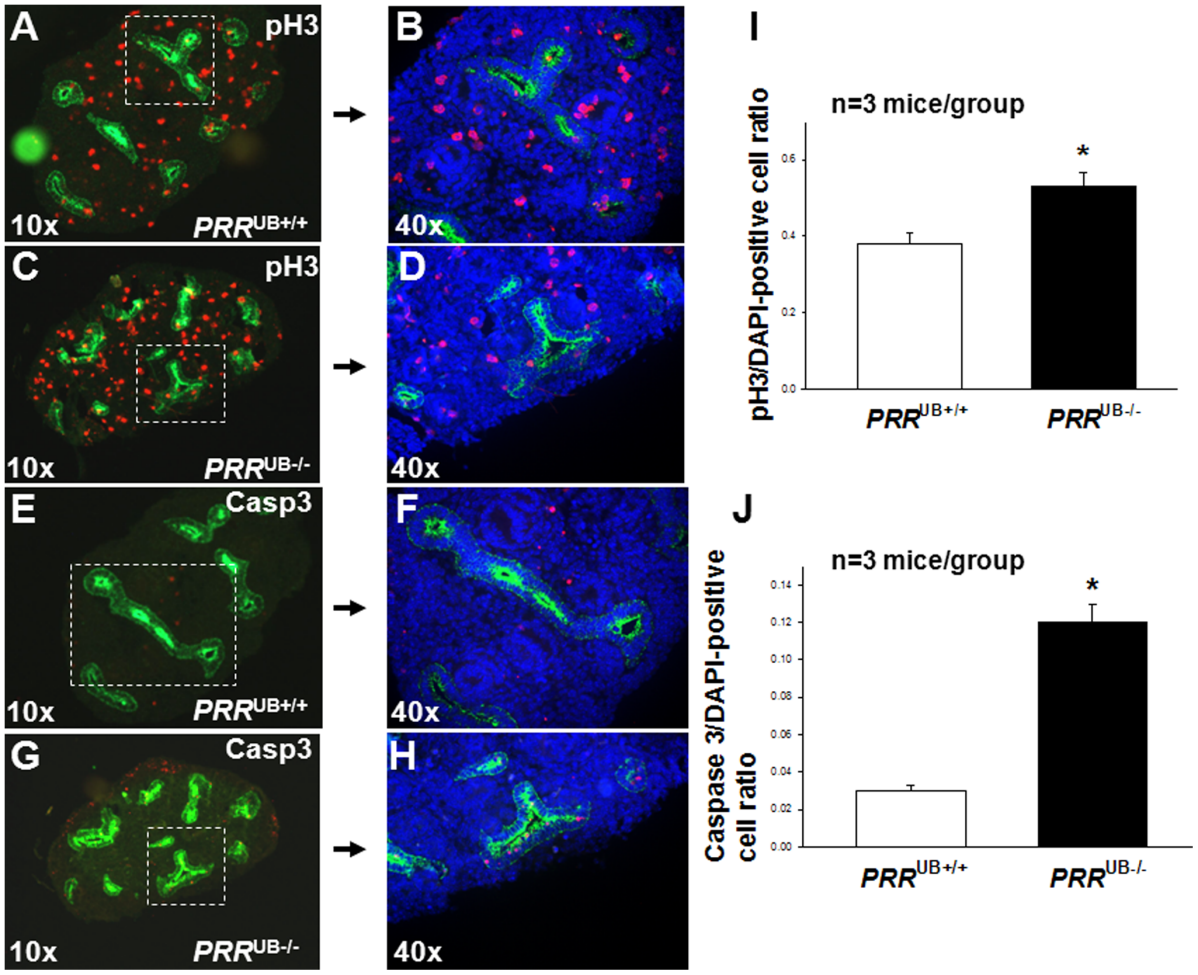
Effect of targeted genetic inactivation of the *PRR* in the UB on UB cell apoptosis and proliferation on E12.5. UBs (A–H) are visualized with anti-pancytokeratin antibody (green). A–D: Proliferating cells are identified by anti-phospho-histone H3 (pH3) antibody (red). E–H: Apoptotic cells are identified by anti-caspase 3 (Casp3) antibody staining (red). All cells in B, D, F and H are identified by DAPI stain (blue). I, J: Bar graphs show the effect of targeted genetic inactivation of the *PRR* in the UB on the ratio of pH3/DAPI-positive (I) and caspase 3/DAPI- positive (J) cells. *p<0.001.

**Figure 5:**
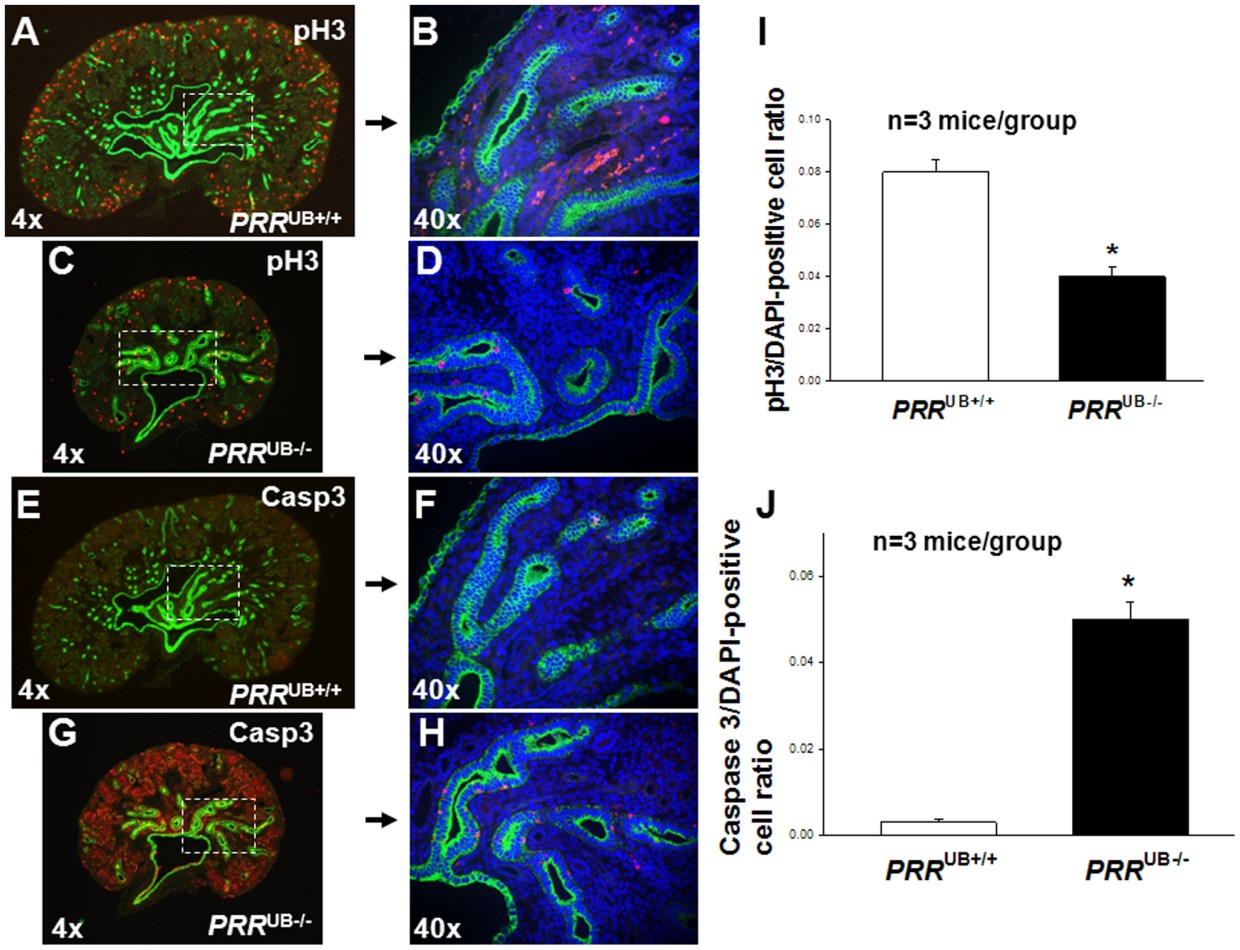
Effect of targeted genetic inactivation of the *PRR* in the UB on collecting duct (CD) cell apoptosis and proliferation on E18.5. A–H: CDs are visualized with anti-pancytokeratin antibody (green). A–D: Proliferating cells are identified by anti-phospho-histone H3 (pH3) antibody (red). E–H: Apoptotic cells are identified by anti-caspase 3 (Casp3) antibody staining (red). All cells in B, D, F and H are identified by DAPI stain (blue). I, J: Bar graphs show the effect of targeted genetic inactivation of the *PRR* in the UB on the ratio of pH3/DAPI-positive (I) and caspase 3/DAPI- positive (J) cells. *p<0.001.

To identify the cellular mechanisms by which *PRR* deficiency in the UB could cause aberrant UB branching morphogenesis, we examined UB cell proliferation and apoptosis in *PRR* ^UB−/−^ and control mice on E12.5. The ratio of both caspase 3-positive apoptotic cells and of pH3-positive proliferating cells in the UB to the total number of DAPI-positive cells per kidney section was increased in *PRR* ^UB−/−^ mice (caspase 3∶0.12±0.01 vs. 0.03±0.003, p<0.01; pH3∶0.53±0.03 vs. 0.38±0.02, p<0.05) compared with control mice (Figure 4). At 18.5, the ratio of apoptotic/DAPI-positive cells was increased (0.05±0.004 vs. 0.002±0.0003, p<0.001) whereas the ratio of proliferating/DAPI-positive cells was reduced (0.04±0.003 vs. 0.08±0.005, p<0.01) in collecting ducts of *PRR* ^UB−/−^ compared to control mice (Figure 5). These findings demonstrate an important role for the PRR in epithelial cell proliferation and survival during UB branching and collecting duct development, and suggest that enhanced UB cell apoptosis may account for UB branching defects observed in *PRR* ^UB−/−^ mice.

### *PRR* Deletion Results in Renal Hypodysplasia

**Figure 6:**
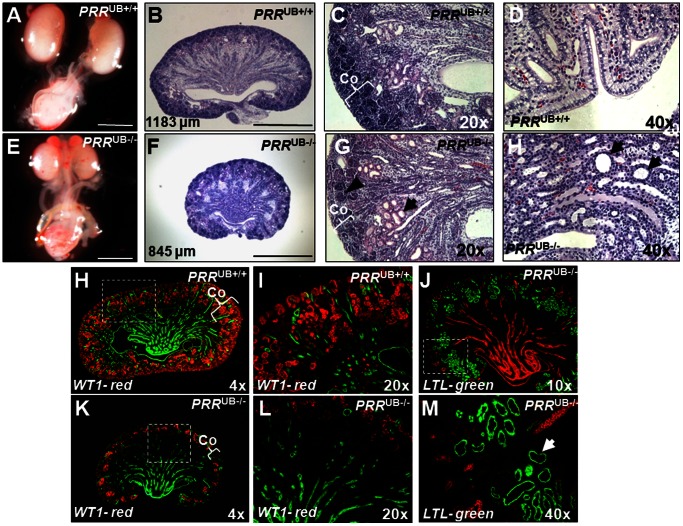
Gross images of P1 urogenital blocks and histological sections of P1 kidneys from control (A–D, H, I) and *PRR* ^UB−/−^ (E–L, J–M) mice. Mutant kidneys (E, F, K) are smaller than control kidneys (A, B, H). Numbers in panels B and F show kidney length. Hematoxylin and eosin-stained sections show the presence of collecting duct cysts (H, arrows), dilated tubules (G, arrow), thin cortex (Co, brace), and decreased number of nephrons (G, arrowhead) in mutant kidneys. H, I, K, L: Kidney sections co-stained with anti-Wilms tumor 1 (WT1, red) and anti-pancytokeratin (green) antibodies show apparent reduction in WT1 staining in *PRR* ^UB−/−^ (K, L) compared with *PRR* ^UB+/+^ (H, I) mice. J, M: Kidney sections of *PRR* ^UB−/−^ mice co-stained with anti-Lotus Tetragonolobus Lectin (LTL, green) and anti-pancytokeratin (red) antibodies show that some of the dilated tubules (M, arrow) are from a proximal tubule origin. I, L, M: High power images of areas shown by dashed line insets in H, J and K. N = 3 mice per genotype. Scale bars (A, B, E, F)- 500 µm.

Dissection of urogenital tracts of newborn mice showed that mutants had smaller kidney size (kidney length: 800±20 vs. 1150±32 µm, p<0.001) (Figure 6). On P1, body weight did not differ in mutant and control mice (4.08±0.11 vs. 4.12±0.13 g, p = 0.82). In contrast, kidney weight (15.6±0.45 vs. 24.5±0.43 mg, p<0.001) and kidney-to-body weight ratio (3.77±0.09 vs. 5.90±0.26 mg/g, p<0.01) was lower in mutants than in controls. Gross histological examination revealed absence of apparent hypoplasia of the renal medulla in mutant mice (Figure 6). The medulla/cortex ratio did not differ in mutant and control P1 kidneys (0.41±0.04 vs. 0.35±0.03, p = 0.2). Detailed histological examination demonstrated the presence of occasional cysts in the collecting ducts, dilation of tubules from a proximal tubule origin, thin cortex and decreased nephron number in mutant compared to control kidneys on P1 (mean number of nephrons per section per kidney: 42±3.5 vs. 85±5.8, p<0.001) (Figure 6). The intensity of WT1 staining was reduced in mutant (Figure 6K, L) compared to control (Figure 6H, I) kidneys on P1 (479153±77199 vs. 2297671±88702 pixels, p<0.001). Reduced nephron endowment observed in newborn mutant mice corresponds with the reduction in UB branching observed during gestation. On P30, body weight did not differ in mutant and control mice (16.4±0.23 vs. 16.9±0.25 g, p = 0.2). In contrast, kidney weight (130±5.8 vs. 280±20 mg, p<0.001) and kidney-to-body weight ratio (7.9±0.23 vs. 16.2±0.6 mg/g, p<0.01) was lower in mutants than in controls.

### *PRR* ^UB−/−^ Kidneys Display Aberrant Terminal Differentiation of the UB Epithelium and Altered Wnt mRNA Expression

**Figure 7:**
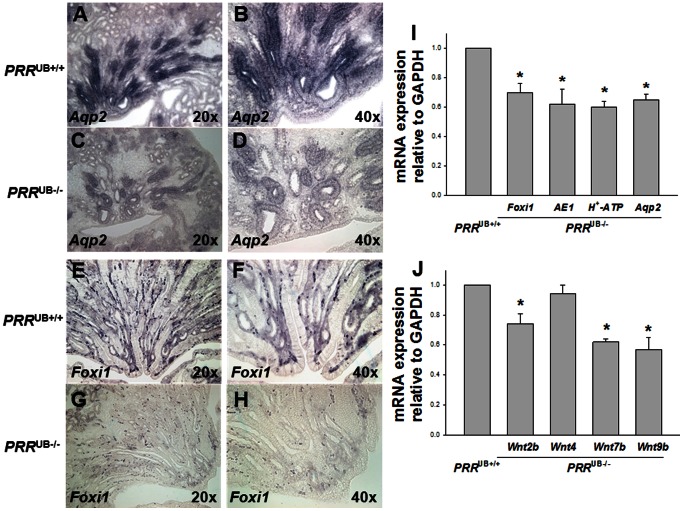
Effect of targeted genetic inactivation of the *PRR* in the UB on *Aqp2, Foxi1, AE1* and H^+^-ATPase subunit *α4* mRNA expression in the mouse metanephros on E18.5. A–H: Representative images of E18.5 section *in situ* hybridization. *PRR* ^UB−/−^ kidneys show apparent decrease in *Aqp2* (C, D) and *Foxi1* (G, H) mRNA expression compared with *PRR* ^UB+/+^ kidneys (A, B, E, F). I: Bar graph showing decreased *Aqp2, Foxi1, AE1* and H^+^-ATPase subunit *α4* mRNA levels in whole E18.5 *PRR* ^UB−/−^ metanephroi as determined by qRT-PCR. J: Bar graph showing decreased *Wnt2b, Wnt7b* and *Wnt9b* mRNA levels in whole E18.5 *PRR* ^UB−/−^ metanephroi as determined by qRT-PCR. *PRR* ^UB+/+^ value is defined as 1. *p<0.001 vs. *PRR* ^UB+/+^.

**Figure 8:**
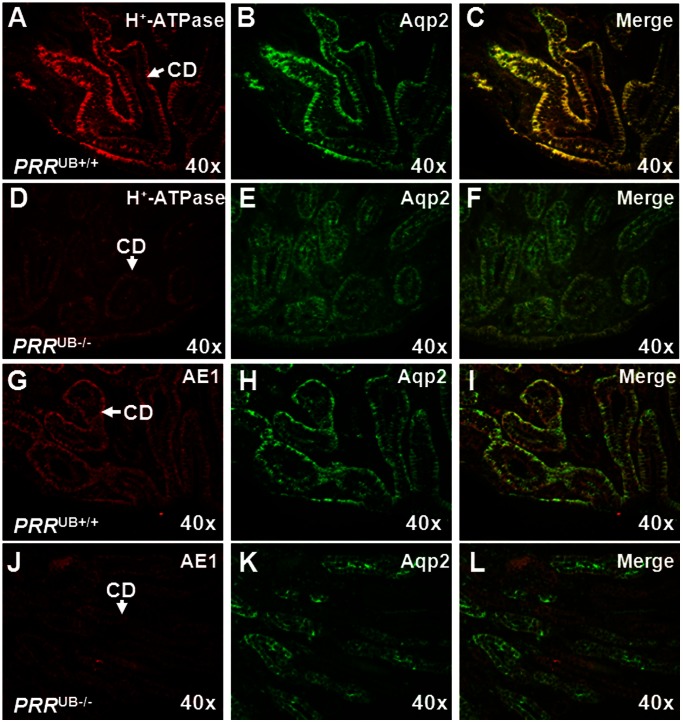
Immunolocalization of H^+^-ATPase subunit α4 and AE1 proteins in the *PRR* ^UB−/−^ and control kidneys on E18.5. A–F: Sections were co-stained with anti-H^+^-ATPase subunit α4 (red) and anti-Aqp2 (green) antibodies. The number of H^+^-ATPase subunit α4-positive collecting duct cells appears to be reduced in mutant (D–F) compared to control (A–C) kidneys. G–L: Sections were co-stained with anti-AE1 (red) and anti-Aqp2 (green) antibodies. The number of AE1-positive collecting duct cells appears to be reduced in mutant (J–L) compared to control (G–I) kidneys. CD- collecting duct.

To determine whether PRR regulates terminal differentiation of the collecting duct cells involved in water permeability and acid-base homeostasis, we examined the expression of winged helix transcription factor Foxi1, chloride-bicarbonate exchanger AE1, α-intercalated cell (α-IC)-specific H^+^-ATPase subunit α4 and water channel Aqp2 mRNA in *PRR* ^UB−/−^ and control mice on E18.5. qRT-PCR showed decreased expression of *Foxi1*, *AE1*, *H^+^-ATPase α4* and *Aqp2* mRNA in mutants (Figure 7). These results were validated by *in situ* hybridization with *Aqp2* and *Foxi1* probes (Figure 7) and by immunohistochemistry with anti-H^+^-ATPase *α*4 and -AE1 antibodies (Figure 8). The number of H^+^-ATPase α4-positive ICs per collecting duct was reduced in mutant compared with control kidneys (3.0±0.28 vs. 10.5±2.0, p = 0.003), indicating that the observed decrease in H^+^-ATPase α4 expression is not due to reduced number of collecting ducts in *PRR* ^UB−/−^ kidneys. Thus, PRR in the collecting duct acts to enhance Foxi1, AE1, H^+^-ATPase subunit α4 and Aqp2 expression levels during terminal differentiation of UB epithelium.

Given that Wnt signaling is prerequisite for proper metanephric development and that PRR is required for both canonical Wnt/*β*-catenin and frizzled (Fz)/planar cell polarity (PCP) signaling in *Drosophila* [26]–[28], we examined whether targeted deletion of UB *PRR* alters *Wnt2b*, *Wnt4, Wnt7b* and *Wnt9b* mRNA expression on E18.5 by whole-kidney qRT-PCR. *Wnt2b*, *Wnt7b* and *Wnt9b* mRNA levels were lower in *PRR* ^UB−/−^ compared to *PRR* ^UB+/+^ kidneys, whereas *Wnt4* expression did not differ (Figure 7). These findings indicate that UB PRR may control UB/collecting duct development, in part, *via* the regulation of Wnt pathway.

### *PRR* Deletion Impairs Kidney Function, Urinary Concentration and Acidification Ability

**Figure 9:**
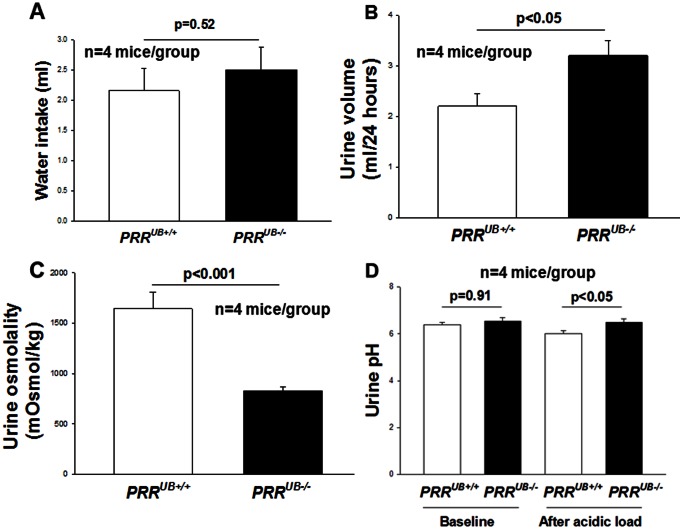
Bar graphs showing water intake, 24-hour urine volume, urine osmolality and urine pH in *PRR* ^UB−/−^ and *PRR* ^UB+/+^ mice on postnatal day P30. Despite no difference in water intake (A), *PRR* ^UB−/−^ mice have increased urine volume (B), decreased urine osmolality (C) and increased urine pH after acidic load compared to *PRR* ^UB+/+^ mice.

To test the functional consequences of the deficiency of the collecting duct *PRR*, we examined renal function, urinary volume, osmolality and pH on P30 during *ad libitum* water intake. Mutant mice had increased levels of serum creatinine on P30 (237±0.13 vs. 59±14 µmol/l, p<0.001). Despite similar water intake (2.5±0.37 vs. 2.2±0.36 ml, p = 0.52), 24-hour urine volumes were higher (3.2±0.32 vs. 2.2±0.24 ml, p<0.05) and urine osmolalities were lower (830±39 vs. 1645±160 mg, p<0.001) in mutants compared with controls (Figure 9). These results demonstrate that PRR in epithelial cells of the collecting duct contributes to the concentration of the urine. Since H^+^-ATPase is important for secretion of protons into the tubular lumen, we next examined the ability of *PRR*-mutant mice to acidify the urine. While baseline urine pH did not differ between *PRR* ^UB−/−^ and *PRR* ^UB+/+^ mice (6.40±0.05 vs. 6.52±0.17, p = 0.51), it was higher following 48 hours of acidic loading with NH_4_Cl in mutants compared with controls (6.51±0.13 vs. 6.02±0.12, p<0.05) (Figure 9). Acidic load resulted in a decrease in urine pH in control (6.40±0.05 vs. 6.02±0.12, p<0.05) and no change in urine pH in mutant (6.52±0.17 vs. 6.50±0.13, p = 0.91) mice. These findings demonstrate a decreased capacity of *PRR* ^UB−/−^ mice to handle an acidic load.

## Discussion

Here, we provide the first *in vivo* evidence demonstrating that PRR is essential for UB branching and collecting duct development. *PRR* ^UB−/−^ mice have profound defects in the UB lineage, including reduced UB branching, occasional cysts in the medullary collecting ducts, which ultimately result in a decreased number of nephrons and marked kidney hypoplasia. These abnormalities are secondary, at least in part, to aberrant apoptosis and proliferation of UB and collecting duct cells, reduced phosphorylation of Erk1/2, decreased expression of *Ret*, *Wnt11*, *Etv4* and *Etv5* in the UB epithelia. Mutant kidneys show decreased expression of *Foxi1*, *AE1*, *H^+^-ATPase α4* and *Aqp2*, revealing that PRR in the collecting duct is required for the proper expression of genes needed for maintaining adequate water and acid-base homeostasis. Finally, mutant mice have polyuria with defects in urinary concentrating and acidification capacity.

A new role for PRR signaling in the UB lineage revealed in *PRR* ^UB−/−^ mice is to stimulate UB branching by controlling *Ret*/*Wnt11* pathway gene expression and signaling *via* Erk1/2. *Ret* and *Wnt11* function in a positive feedback loop to promote UB branching *via* induction of proliferation and migration of the UB tip cells by maintaining a balance between appropriate expression of glial-derived neurotrophic factor (*GDNF*) in the mesenchyme and of *Ret*/*Wnt11* in the UB tips [29], [30]. The Pea3 family of *Ets* transcription factors *Etv4*/*Etv5* and Erk1/2 are indispensable for transmission of Ret signals to regulate UB branching [31], [32]. Thus, decreased expression of *Ret*, its downstream targets, *Wnt11*, *Etv4/Etv5*, and reduced Erk1/2 phosphorylation in the UB of *PRR* ^UB−/−^ mice are probably a major driving force behind UB branching defects. The observed increase in cell apoptosis in UB branches in *PRR* ^UB−/−^ mice is consistent with the known function of *Ret* signaling to promote UB cell survival [33]. Since targeted inactivation of the *PRR* in mouse cardiomyocytes causes cardiomyocyte apoptosis, the effect of *PRR* depletion on UB-derived collecting duct cell survival observed in the present study may be, in part, *Ret*-independent [10]. In addition to aberrant cell proliferation and apoptosis, defects in collecting duct planar cell polarity/oriented cell division may explain presence of occasional collecting duct cysts in *PRR* mutants. This possibility is supported by the observations that treatment of *Xenopus* embryos with anti-PRR morpholinos causes a short body axis, smaller head size and a broader expression domain of Xnot, a hallmark of impaired convergent extension movements [26]. In addition, *Drosophila* PRR interacts biochemically with Fz receptor, which is required for PCP signaling, in human embryonic kidney HEK293T cells [26]. Presence of marked renal hypoplasia in *PRR* ^UB−/−^ mice in the absence of changes in medulla/cortex ratio suggests that the observed phenotype is due predominantly to a proportional decrease in nephron endowment resulting from reduction in UB branching rather than to decreased elongation of medullary collecting ducts. Consistent with this interpretation, reductions in UB branching and nephron number are characteristic of human renal hypodysplasia, a form of CAKUT observed in 1 in 400 births [34].

Regulation of acid-base homeostasis by the kidney is essential for health. In the collecting duct, *α*-ICs secrete protons into the tubular lumen through apical membrane H^+^-ATPase functionally coupled to the basolateral membrane chloride-bicarbonate transporter anion exchanger 1 (AE1) [35]. Differentiation of ICs requires the winged helix transcription factor Foxi1, as *Foxi1* ^UB−/−^ mice develop distal renal tubular acidosis (dRTA) due to absence of ICs [8]. Given that PRR is an accessory subunit of the vacuolar proton pump H^+^-ATPase, which is expressed at the apical surface of collecting duct *α*-ICs [9], [11], [35], we strongly suspected that *PRR* deletion in the collecting duct would lead to aberrant H^+^-ATPase expression Indeed, expression of α-IC-specific H^+^-ATPase subunit α4 was reduced in *PRR* mutants. Reduction in H^+^-ATPase subunit α4 levels was accompanied by decreased expression of Foxi1 and AE1. Since Foxi1, reported to mediate differentiation of ICs from epithelial precursor UB cells, is necessary for expression of the H^+^-ATPase subunit α4 and AE1 in the collecting duct, decreased expression of ATPase subunit α4 and AE1 observed in this study is likely secondary to a reduction in Foxi1 levels [8], [36]. Thus, PRR is a direct or indirect regulator of gene expression in α-ICs. The importance of these findings is underscored by the fact that disruption of *α*-IC function due to mutations in genes encoding H^+^-ATPase α4 subunit or AE1 results in inheritable forms of dRTA in humans [37], [38]. ICs are present in the collecting duct in a random distribution among the majority principal cells (PCs). To determine whether *PRR* deletion in the collecting duct alters terminal differentiation of PCs, we examined expression of water channel Aqp2. *Aqp2* mutations in humans cause nephrogenic diabetes insipidus, a disease where water resorption by the collecing duct is eliminated [39]. Abundance of Aqp2 mRNA and protein levels was diminished in *PRR* ^UB−/−^ collecting ducts. Hence, PRR signaling may be also important in terminal differentiation and function of PCs. We found apparently normal localization of Aqp2 protein at the apical surface of PCs. Thus, PRR in the collecting duct does not appear to affect trafficking of Aqp2 *in vivo*.

To examine the impact of targeted *PRR* deletion on collecting duct function, we analyzed renal concentrating and acidification ability in *PRR* ^UB−/−^ and *PRR* ^UB+/+^ mice. Despite the presence of grossly morphologically normal, although relatively smaller compared with *PRR* ^UB+/+^ mice, medulla and similar water intake, *PRR* ^UB−/−^ mice were polyuric and had impaired urine-concentrating ability. These findings suggest that PRR regulates the urinary concentrating mechanism through direct affect on collecting duct epithelium *via* downregulation of Aqp2, a major determinant of urinary concentrating capacity [6]. Given that expression of IC genes important for urinary acidification was decreased in the collecting duct epithelium of *PRR* ^UB−/−^ mice, we hypothesized that mutants had reduced ability to acidify the urine. Indeed, our findings revealed a decreased capacity to acidify the urine after acidic load in *PRR* mutants. Thus, collecting duct PRR is important in establishment and function of collecting duct cells involved in acid-base homeostasis. These features of the reduced H^+^-ATPase expression, coupled with a decreased ability to secrete protons into the urine in response to acidic loading, in *PRR* mutants are in agreement with the reported role of the PRR in podocytes, where targeted *PRR* depletion downregulates the expression of the V_0_ c subunit of H^+^-ATPase resulting in an increased vesicular pH [40], [41].

In summary, we demonstrate that UB/collecting duct PRR is essential for kidney morphogenesis and acquisition of function by collecting duct cells involved in water and acid-base homeostasis. Thus, PRR is a potential candidate for future genetic screening studies in patients with renal hypodysplasia or disorders of water resorption and dRTA.
